# A lithium-containing biomaterial promotes chondrogenic differentiation of induced pluripotent stem cells with reducing hypertrophy

**DOI:** 10.1186/s13287-020-01606-w

**Published:** 2020-02-21

**Authors:** Yaqian Hu, Lei Chen, Yi Gao, Pengzhen Cheng, Liu Yang, Chengtie Wu, Qiang Jie

**Affiliations:** 1 Department of Orthopedic Surgery, Honghui Hospital, College of Medicine, Xi’an Jiaotong University, Xi’an, 710049 People’s Republic of China; 2 Institute of Orthopedic Surgery, Xijing Hospital, Fourth Military Medical University, Xi’an, 710032 People’s Republic of China; 3 State Key Laboratory of High Performance Ceramics and Superfine Microstructure, Shanghai Institute of Ceramics, Chinese Academy of Science, Shanghai, 200050 People’s Republic of China

**Keywords:** iPSCs, Li_2_Ca_4_Si_4_O_13_ bioceramic, Chondrocytes

## Abstract

**Background:**

Induced pluripotent stem cells (iPSCs) exhibit limitless pluripotent plasticity and proliferation capability to provide an abundant cell source for tissue regenerative medicine. Thus, inducing iPSCs toward a specific differentiation direction is an important scientific question. Traditionally, iPSCs have been induced to chondrocytes with the help of some small molecules within 21–36 days. To speed up the differentiation of iPSCs, we supposed to utilize bioactive ceramics to assist chondrogenic-induction process.

**Methods:**

In this study, we applied ionic products (3.125~12.5 mg/mL) of the lithium-containing bioceramic (Li_2_Ca_4_Si_4_O_13_, L2C4S4) and individual Li^+^ (5.78~23.73 mg/L) in the direct chondrogenic differentiation of human iPSCs.

**Results:**

Compared to pure chondrogenic medium and extracts of tricalcium phosphate (TCP), the extracts of L2C4S4 at a certain concentration range (3.125~12.5 mg/mL) significantly enhanced chondrogenic proteins Type II Collagen (COL II)/Aggrecan/ SRY-Box 9 (SOX9) synthesis and reduced hypertrophic protein type X collagen (COL X)/matrix metallopeptidase 13 (MMP13) production in iPSCs-derived chondrocytes within 14 days, suggesting that these newly generated chondrocytes exhibited favorable chondrocytes characteristics and maintained a low-hypertrophy state. Further studies demonstrated that the individual Li^+^ ions at the concentration range of 5.78~23.73 mg/L also accelerated the chondrogenic differentiation of iPSCs, indicating that Li^+^ ions played a pivotal role in chondrogenic differentiation process.

**Conclusions:**

These findings indicated that lithium-containing bioceramic with bioactive specific ionic components may be used for a promising platform for inducing iPSCs toward chondrogenic differentiation and cartilage regeneration.

## Background

Articular cartilage regeneration has always been a tricky problem in the field of skeleton repair. It is almost impossible for damaged cartilage to naturally heal because of its avascularity and absence of stem cell migration [1]. Produced by chondrocytes, extracellular matrix components trap chondrocytes and prevent them from migrating and repairing once the cartilage is damaged. Clinical approaches treating cartilage defects usually resort to autologous cartilage or endogenous mesenchymal stem cells (MSCs) [2]; however, these approaches suffer from a limited amount of autologous cartilage and poor chondrogenic differentiation ability of aged MSCs. Therefore, it is important to generate functional chondrocytes from an adequate source.

In recent years, iPSCs were used as potentially unlimited cells for tissue regeneration. The iPSCs were considered as an attractive cell source for cartilage repair owing to their abundance, autologous nature, and potentiality to generate adequate chondrocytes rather than other cell sources [3]. While there was currently no generally accepted efficient protocol for differentiation chondrocytes from iPSCs [4], producing embryoid bodies (EBs) or MSC-like cells from iPSCs before chondrocytes differentiation were reported as regular methods by previous studies [5–7]. Nevertheless, these methods were time-consuming for multiple differentiation steps and would cause adverse effects on the urgency of clinical cartilage repair. Therefore, there were a few attempts to apply biomaterials for facilitating cartilage repair in a combination of iPSCs in vivo [2, 8, 9]. However, the direct use of undifferentiated iPSCs-biomaterials composites for cartilage defects repair is not completely safe because of the adventure of teratoma formation. Thus, if iPSCs can be rapidly induced into chondrocytes by means of biomaterials in vitro before implanted in cartilage defects, it will be of great significance for the urgency and safety of clinical cartilage repair. Nevertheless, to our knowledge, it is unclear whether the biomaterial can observably promote chondrogenic differentiation of iPSCs in vitro.

In order to solve these doubts, we synthesized a new L2C4S4 bioceramics which was proven to promote in vivo repair of osteochondral defects in our previous study [10]. In view of the advantageous stimulatory effects of the ionic products from L2C4S4 on chondrogenesis in vitro, we supposed that ionic products of this bioceramics may also biologically facilitate chondrogenic differentiation of iPSCs.

In this study, serial dilutions of extracts of L2C4S4 powders (3.125~12.5 mg/mL) were applied to the directly chondrogenic induction process of iPSCs, the TCP powders were served as a control. Various chondrocytes indicators were obtained and analyzed after 14 days of chondrogenic differentiation. Compared with pure chondrocyte-inducing medium or the extracts of TCP, the extracts of L2C4S4 accelerated the chondrogenic differentiation of iPSCs and significantly prevented the hypertrophy of newly derived chondrocytes. Furthermore, when individual Li^+^ ions at different concentrations (5.78~23.73 mg/L) were applied in the chondrogenic induction of iPSCs in the same way, it also showed similar results. From the above, L2C4S4 represented a promising biomaterial for directly chondrogenic differentiation of iPSCs within a short time, and the Li^+^ ions in this bioceramic made an important contribution to this differentiation process.

## Methods

### Human iPSCs culture and identification

Human iPSCs were established with the help of Shenzhen Cell Inspire Biotechnology Company and cultured in mTeSR1 medium at 37 °C in a humidified CO_2_ incubator. The medium was changed every day. The iPSCs were passaged to Matrigel-coated polystyrene plates. Before being conduced in the experiment, the iPSCs underwent a variety of tests on cell pluripotency.

Alkaline phosphatase staining: undifferentiated iPSCs were washed with PBS and fixed with 4% paraformaldehyde for 5 min. Staining was done using an alkaline phosphatase detection kit (Beyotime Biotechnology, People’s Republic of China). Cells were washed with PBS and surveyed under the microscope.

EB induction and three germ layers differentiation: For EB formation, iPSCs were added onto ultra-low-attachment plates (Corning, USA) with a concentration of 3000/20ul. After 7 days of EB formation, EBs were cultured in α-MEM medium with 10% FBS in 6-well plate for 7 days to differentiate into three germ layer cells.

### The cell proliferation assay

L2C4S4- and TCP-graded extracts in mTeSR1 medium were used to culture human iPSCs for 7 days in 6-well plates. A nuclear protein Ki67 which shows cell division was detected to evaluate the cell proliferation in different L2C4S4 and TCP extracts. If the fluorescence of a nucleus (blue) and ki67 (red) co-localized, the cell was regarded as a positive cell which is proliferating. The positive cell number of each group was statistically present as mean ± the standard error of the mean (SEM) and *p* values of significance is calculated by Student’s *t* test (tails = 2, two-sample unequal variance). *p < 0.05; **p < 0.01, and ***p < 0.001, ns is no significance with *p* > 0.05.

### Preparation of the extracts of L2C4S4 and TCP powders

The L2C4S4 bioceramic powders were synthesized and characterized as previously reported [10]. Pure TCP powders were prepared as the control. Extracts of the L2C4S4 and TCP powders were prepared following the protocol of the International Standard Organization (ISO/EN 10993-5). Briefly, steam sterilization method was utilized to sterilize the L2C4S4 and TCP powders before soaked in serum-free chondrogenic differentiation medium (MCDM; SclenCell, USA) or mTeSR1 medium (STEMCELL Technologies, Canada) at the concentration of 200 mg/mL. After incubated at 37 °C for 24 h, the mixtures were centrifuged, and the supernatants were collected. The original extracts were sterilized using a 0.2 μm filter. Subsequently, serial dilutions of extracts (12.5, 6.25, and 3.125 mg/mL) were prepared using MCDM or mTeSR1 medium for further cell culture experiments. The ionic concentrations of Ca, Li, and P in the graded extracts were calculated by inductively coupled plasma atomic emission spectrometry (ICP-AES, 715-ES, Varian, USA). The medium without the addition of material extracts was used as a blank control.

### Chondrocyte spheres induction with extracts of L2C4S4 and TCP

The iPSCs were dissociated to a single cell suspension by Cell Dissociation Buffer (Gibco, USA) and then diluted to a final concentration of 3*10^5^/20ul. Each 20 μl of the cell suspension was added to a low-adherence 24-well plate and cultured in the incubator at 37 °C for 3 h to form a sphere. 0.7 mL MCDM with serial dilutions of extracts (12.5, 6.25 and 3.125 mg/mL) was added to each well slowly. Cells were cultured as non-adherent spheres for 14 days. The medium was changed every other day.

### Chondrocyte spheres induction with LiCl

Ionic concentrations in graded extracts of L2C4S4 and TCP were calculated by inductively coupled plasma atomic emission spectrometry. To mimic Li^+^ ions concentrations in L2C4S4 extracts, 5.78~23.73 mg/L Li^+^ ions were prepared of LiCl (China National Pharmaceutical Group Corporation, People’s Republic of China) and applied in MCDM to culture iPSCs. MCDM without any extracts was served as a control. The iPSCs were dissociated and diluted to a final concentration of 3*10^5^/20ul. Each 20 μl of the cell suspension was cultured in the incubator for 3 h to form a sphere. 0.7 mL MCDM with serial dilutions of Li^+^ ions (5.78~23.73 mg/L) was added to each well. Cells were cultured as non-adherent spheres for 14 days. The medium was changed every other day.

### Immunofluorescence

For immunofluorescence, frozen sections of chondrocyte spheres were washed with PBS for three times, these cell slices were permeabilized with cold 0.2% Triton X-100 (Sigma, USA) in PBS for 5 min. A step of enzymatic antigen retrieval with 0.1% Trypsin in PBS was performed prior to block for 1 h. After blocking, antibodies against Aggrecan (1:300; Abcam, UK), COL II (1:300; Abcam), SOX9 (1:200; Affinity), MMP13 (1:200; Affinity), COL X (1:500; Abcam), NANOG (1:300; Abcam), OCT4 (1:300; Abcam), TRA-1-60(1:300; Abcam) and Ki67 (1:300; Abcam) were added overnight. The following day, cells were washed three times with PBS and then incubated at 37 °C for 1 h with Alexa 594-conjugated goat anti-rabbit secondary antibody (1:300; Abcam) or Alexa 488-conjugated goat anti-mouse secondary antibody (1:300; Abcam). The cell nucleus was counterstained with DAPI (Beyotime, People’s Republic of China).

### Microscopy and statistical analysis

Fluorescent microscopy was performed on an Olympus microscope; images were taken under × 40 objective. In order to compare the differences in immunofluorescence intensity between the groups intuitively, the average fluorescence intensity of the indicated proteins in the view region was analyzed using the Image-Pro Plus 6.0 system. The quantification data were statistically present as mean ± SEM. *p* values of significance is calculated by Student’s *t* test (tails = 2, two-sample unequal variance): *p < 0.05, **p < 0.01, ***p < 0.001, ns is no significance with *p* > 0.05.

### Real-time polymerase chain reaction

To evaluate the mRNA transcript levels of chondrocytes specific genes (Col2a1, Aggrecan, Sox9, Col10a1, Mmp13, and Ihh), stem cell-specific genes (Oct4, Nanog, and Sox2), and three germ layer genes (Nestin, Map 2, Desmin, Msx1, and Sox17), chondrocyte spheres, iPSCs, and EBs were processed for total RNA extraction by using an RNAprep Micro Kit (TaKaRa, Japan) at 14 days or 7 days. The concentration of RNA was determined with an RNA analyzer (Quawell, USA). The cDNA was prepared with PrimeScript RT Master Mix (TaKaRa, Japan). RT-PCR was performed by using SYBR Green QPCR Master Mix (TaKaRa, Japan) with a Light Cycler apparatus (Bio-Rad, USA). Cycle conditions were as follows: activation of HotStarTaq DNA polymerase/inactivation of reverse transcriptase at 95 °C for 30 s; and 39 cycles of 95 °C for 5 s, and 60 °C for 30 s. The relative expression level of each target gene was calculated by using the 2^−ΔΔCt^ method. All of the primers’ information was provided in Table. S1. Results were repeated for three independent biological replicates. The RT-PCR data were statistically present as mean ± SEM and *p* values of significance were calculated by Student’s *t* test (tails = 2, Two-sample unequal variance) in Excel: *p < 0.05, **p < 0.01, ***p < 0.001, ns is no significance with *p* > 0.05.

## Results

### Characterization of iPSCs generated by cellular reprogramming

**Fig. 1:**
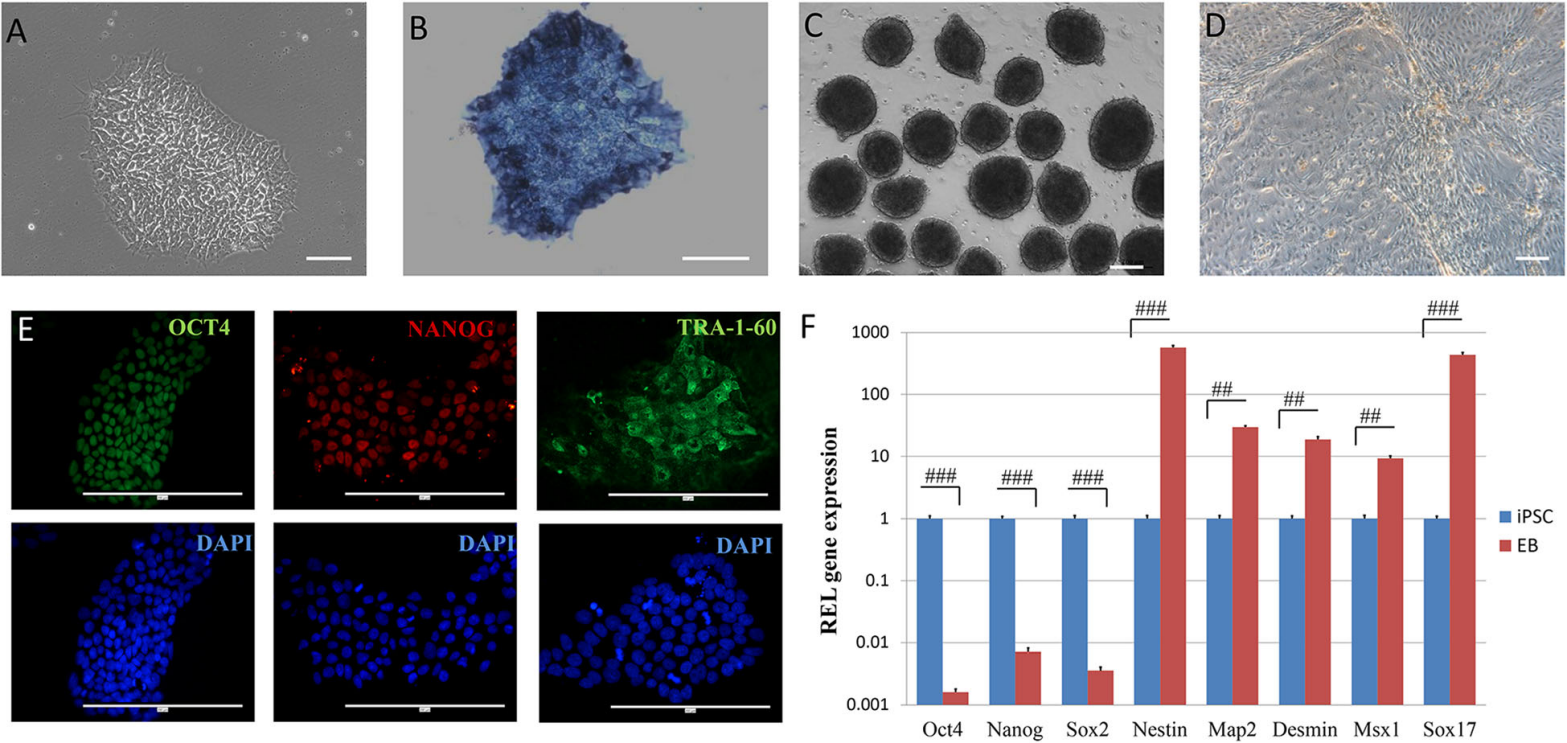
The iPSCs identification. The iPSCs showed embryonic stem cell-like morphology during expansion (**a**) and exhibited positive alkaline phosphatase staining (**b**). EBs were formed (**C**) and able to differentiate into three germ layer cells (**d**). The iPSCs displayed obvious immunofluorescence of pluripotent proteins OCT4, NANOG, and TRA-1-60 (**e**). The expression of pluripotent genes (Oct4, Nanog, Sox2) and three germ layers genes (Nestin, Map 2, Desmin, Msx1, Sox17) were assessed in iPSCs and EBs (**f**). Scale bar, **a** and **b** 100 μm; **c**–**e** 200 μm. Data presented as mean ± SEM. ^##^*p* < 0.01; ^###^*p* < 0.001

Human iPSCs were generated by cellular reprogramming and cultured in mTeSR1 medium at 37 °C in a humidified CO_2_ incubator. With several passages after cell thawing, iPSCs showed embryonic stem cell-like morphology during an expansion (Fig. 1a) and exhibited positive alkaline phosphatase staining (Fig. 1b). The iPSCs also displayed obvious immunofluorescence of pluripotent proteins OCT4, NANOG, and TRA-1-60 in their nucleus and cytoplasm (Fig. 1e). After culturing on ultra-low-attachment plates with a concentration of 3000/20 μl, the iPSCs formed a large number of EBs with smooth edges and uniform sizes (Fig. 1c), and these EBs could generate cells of three germ layers after cultured in 6-well plate for 7 days (Fig. 1d). The expression of pluripotency genes (Oct4, Nanog, Sox2), ectoderm genes (Nestin, Map 2), mesoderm genes (Desmin, Msx1), and endoderm gene (Sox17) were also assessed by RT-PCR in iPSCs and EBs. The expression level of each gene in iPSCs group was set as 1. Compared to iPSCs, the expression of pluripotency genes had obviously declined (*p* < 0.001) while the genes of the three germ layers had significantly risen (*p* < 0.01) in EBs (Fig. 1f), which indicated great potential for multi-directional differentiation of the cells. Together, these data suggested that the iPSCs exhibited the genuine characteristic of pluripotency.

### L2C4S4 helps keep the proliferation and pluripotency of iPSCs

**Fig. 2:**
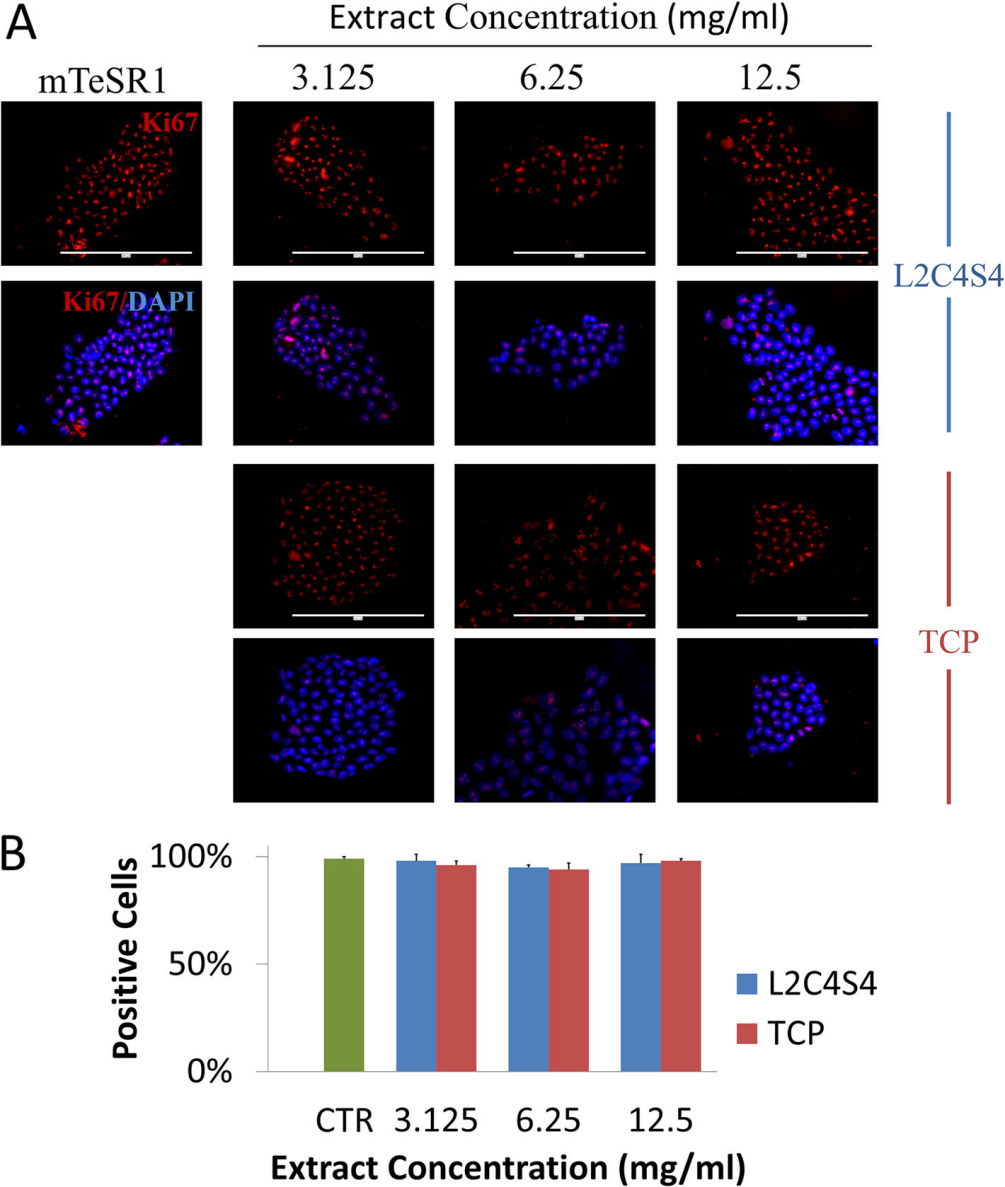
Effects of L2C4S4 extracts on proliferation of iPSCs. **a** Immunofluorescence of Ki67 in different groups. **b** The ratio of positive cells of Ki67. Scale bar = 200um. CTR: mTeSR1 medium without extracts. Data presented as mean ± SEM

Before implementing chondrogenic differentiation with extracts of L2C4S4, we explored their effects on iPSCs proliferation to determine whether they were harmful to iPSCs. Serial dilutions of extracts (12.5, 6.25, and 3.125 mg/mL) of L2C4S4 and TCP were prepared with mTeSR1 medium. The iPSCs were cultured in this medium for 7 days and then underwent proliferation detection. Through fluorescence detection of proliferation marker Ki67, we found that almost every nucleus was labeled in L2C4S4-treated cells at an altered concentration (3.125~12.5 mg/mL), suggesting that all the cells were proliferating. The control group and TCP-treated cells also showed similar proliferation (Fig. 2a). In order to statistically compare the differences between the groups, we defined these ki67-stained cells as positive cells and counted the percentage of positive cells in each group. There was no statistical difference between the groups (*p* > 0.05, Fig. 2b). The iPSCs maintained strong growth momentum after cultured in different concentrations of L2C4S4 extract. This revealed a strong affinity of L2C4S4 without any toxicity to the iPSCs and was important for the subsequent differentiation process.

**Fig. 3:**
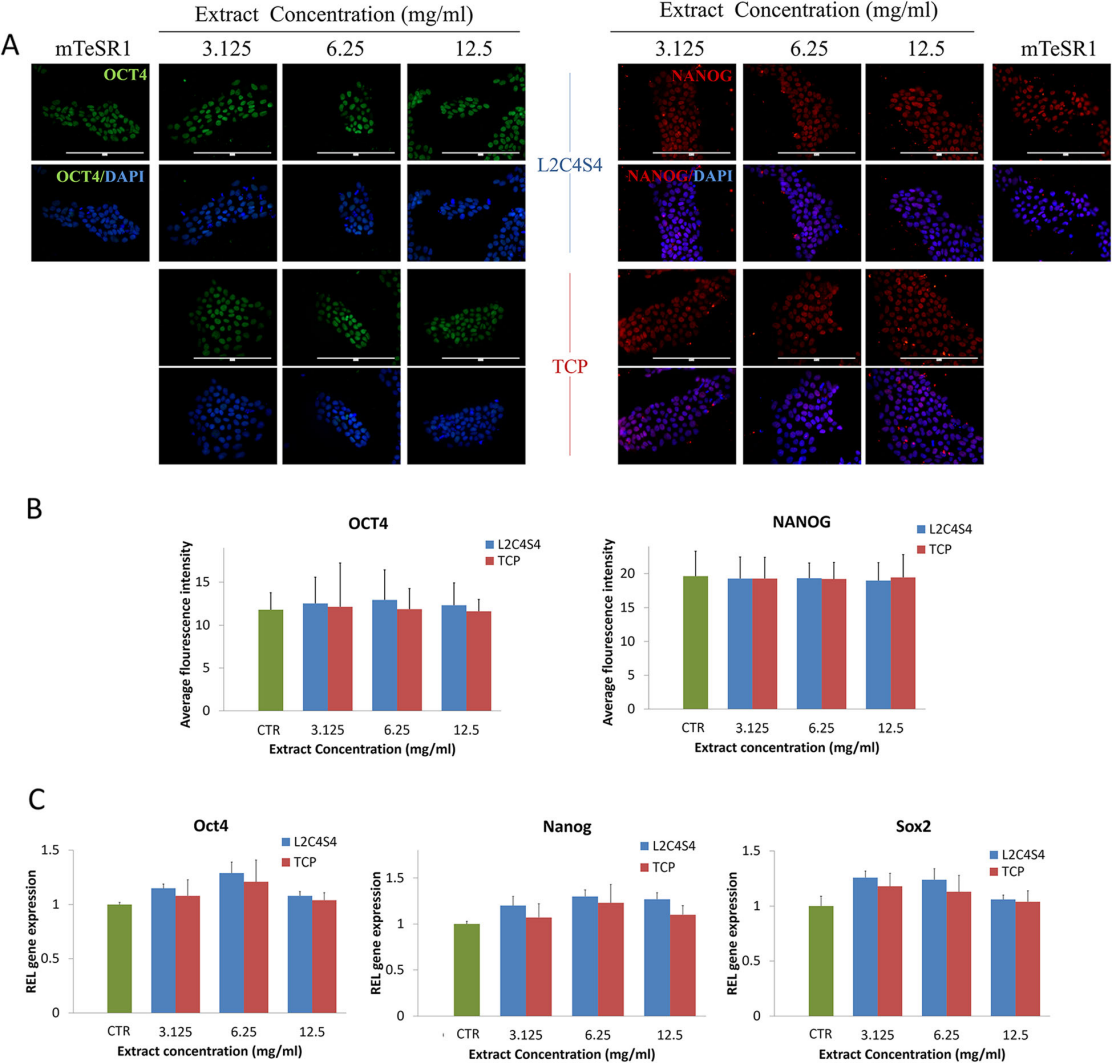
Effects of L2C4S4 extracts on iPSCs pluripotency. **a** Immunofluorescence of pluripotency proteins OCT4 and NANOG. **b** The average fluorescence intensity of OCT4 and NANOG proteins in each group, *n* = 5. **c** RT-PCR of pluripotency genes Oct4, Nanog, and Sox2, *n* = 3. Scale bar = 200um. Data presented as mean ± SEM. CTR: mTeSR1 medium without any extracts

In addition, in order to figure out whether the L2C4S4 can promote spontaneous differentiation of iPSCs, we also carried out pluripotency analyses under the same culture condition. Both of pluripotency proteins OCT4 and NANOG showed high fluorescence level in the nucleus in each group of L2C4S4 and TCP (Fig. 3a). The average fluorescence intensity of these two proteins was analyzed by the Image-Pro Plus 6.0 system. There was no statistical difference between the groups (*p* > 0.05, Fig. 3b). In addition, to explore the effects of L2C4S4 on iPSCs’ pluripotency, RT-PCR analysis of pluripotency genes (Oct4, Nanog, and Sox2) was conducted. There was also no statistical difference between each group (*p* > 0.05, Fig. 3c). These results indicated that the addition of L2C4S4 extracts to the iPSCs medium did not change the pluripotency of iPSCs.

### Beneficial effects of L2C4S4 on the maintaining of chondrocyte spheres

**Fig. 4:**
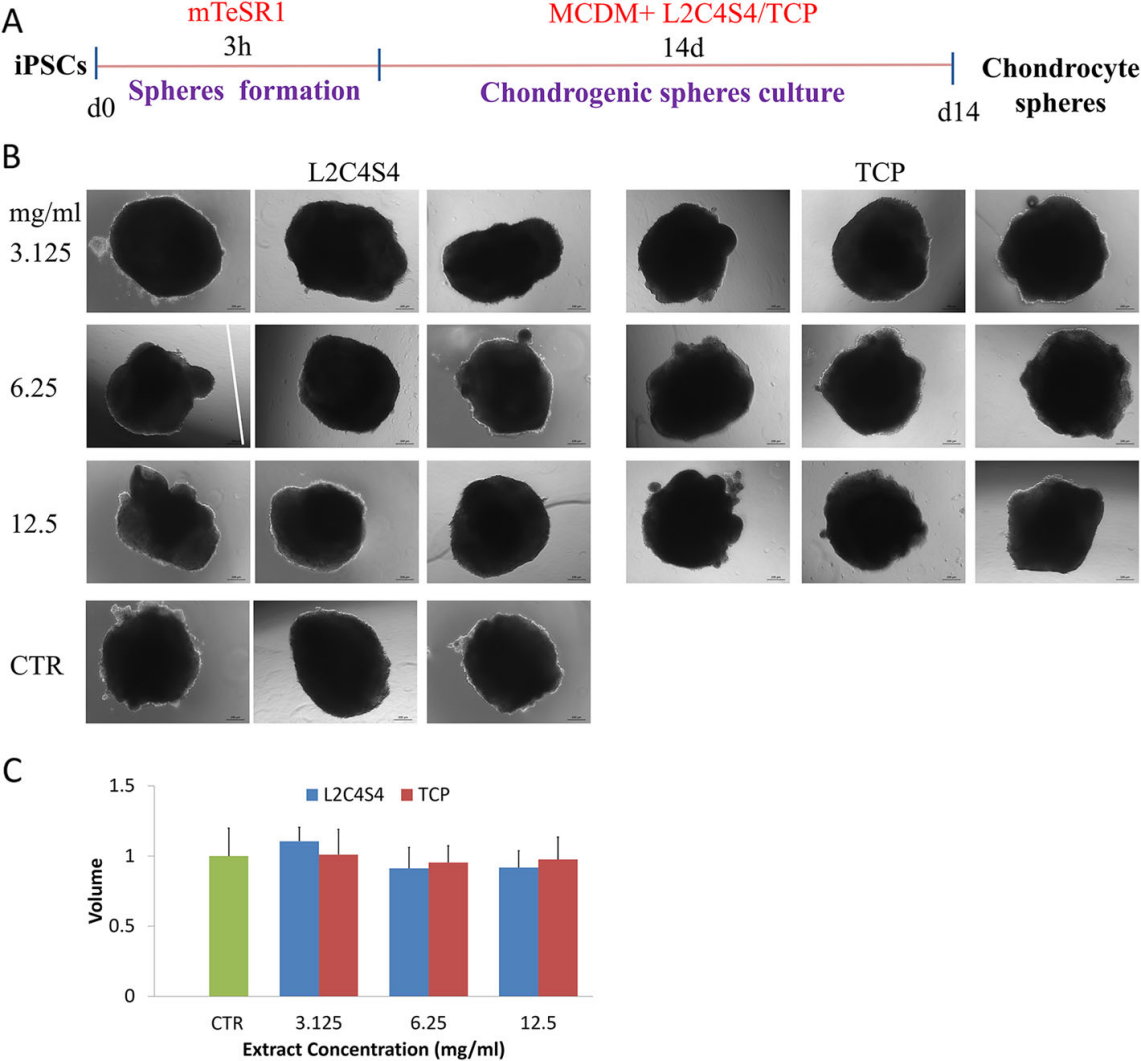
Effects of L2C4S4 extracts on maintaining chondrocyte spheres. **a** Chondrogenic induction strategy. **b** The morphology of chondrocyte spheres in different groups, *n* = 3. **c** The volume of chondrocyte spheres in different groups, *n* = 3, data presented as mean ± SEM. Scale bar = 200 μm. CTR: MCDM without any extracts

The chondrogenic differentiation medium (MCDM) with different dilutions of L2C4S4/TCP extracts was prepared for the chondrogenic differentiation of human iPSCs. The iPSCs were dissociated and diluted to form spheres for 3 h and then cultured as non-adherent spheres in these medium for chondrogenic differentiation for 14 days. Chondrocyte spheres cultured in MCDM without extracts was served as a control. The timeline is shown in Fig. 4a. Each chondrocyte sphere was photographed with a microscope. Similar to control and TCP, altered concentration (3.125~12.5 mg/mL) of L2C4S4 extracts promoted the formation of well-formed spheres (Fig. 4b), which indicated that the L2C4S4 could help maintain normal architecture morphologically of iPSCs-derived chondrocyte spheres.

Volumes of chondrocyte spheres were measured through Image-Pro Plus 6.0 software. To calculate the relative volume of chondrocyte spheres, the value of the control group was set as 1. The volume of the sphere in each group was corrected according to the control group. As showed in Fig. 4c, the volumes of iPSCs-derived chondrocyte spheres in L2C4S4-treated cells were comparable to the control and TCP-treated cells. There was also no statistical difference between each group (*p* > 0.05). It demonstrated that L2C4S4 did not impact the chondrocyte spheres formation in an appropriate size.

### The conducive stimulatory effects of L2C4S4 on chondrogenic differentiation of iPSCs

**Fig. 5:**
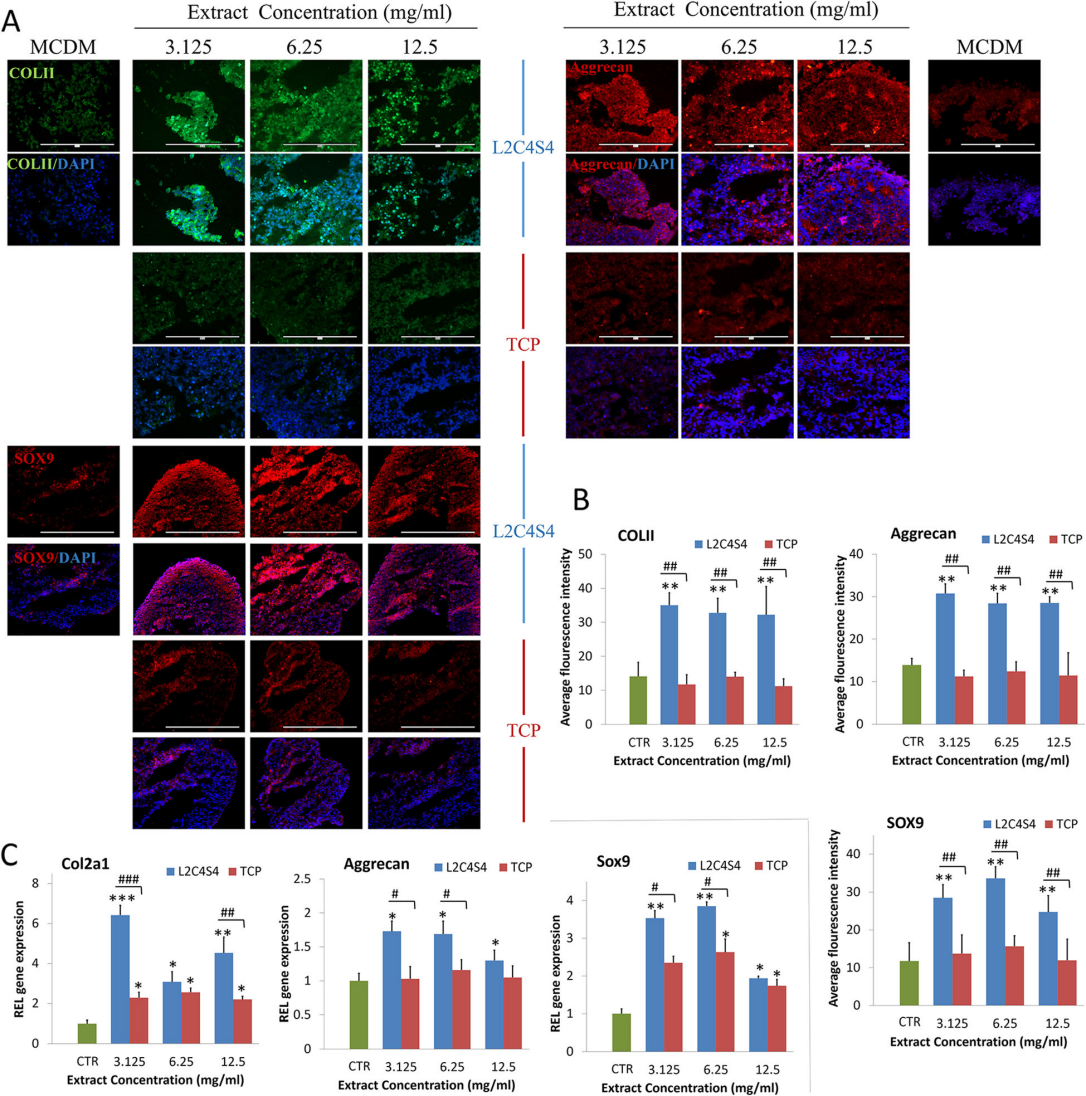
The chondrogenic differentiation of iPSCs with different concentrations of L2C4S4/TCP extracts in 14 days. **a** Immunofluorescence of chondrocytes specified proteins COL II, Aggrecan, and SOX9. **b** The average fluorescence intensity of COL II, Aggrecan and SOX9 proteins in each group, *n* = 5. **c** RT-PCR of chondrogenic genes Col2a1, Aggrecan, and Sox9, *n* = 3. Data presented as mean ± SEM. Scale bar = 200 μm. CTR: MCDM without any extracts. ^#^*p* < 0.05, ^##^*p* < 0.01, ^###^*p* < 0.001, *p < 0.05, **p < 0.01, ***p< 0.001

The iPSCs were cultured in MCDM with different dilutions (3.125~12.5 mg/mL) of L2C4S4 or TCP extracts for 14 days. Expression of chondrocytes specified proteins COL II, Aggrecan, and SOX9 were evaluated by immunofluorescence in chondrocyte spheres. Immunofluorescence findings showed abundant COL II, Aggrecan, and SOX9 massively gathered in the intercellular and intracellular areas of L2C4S4-treated cells while TCP-treated cells and the control group showed a small amount of these proteins’ expression (Fig. 5a). The average fluorescence intensity in each group was analyzed by software Image-Pro Plus 6.0 software. Levels of COL II, Aggrecan, and SOX9 proteins in L2C4S4-treated cells were more than twice as high as the other two groups (Fig. 5b, *p* < 0.01).

Chondrogenic gene expression was also evaluated by RT-PCR on day 14 of chondro-induction. Expression of Col2a1, Aggrecan, and Sox9 genes in L2C4S4-treated cells was significantly enhanced at the concentration range of 3.125~12.5 mg/mL (*p* < 0.05) as compared to control group (Fig. 5c). As compared to TCP-treated cells, the Col2a1 gene was significantly enhanced by L2C4S4 extracts at the concentration of 3.125 mg/mL (*p* < 0.001) and 12.5 mg/mL (*p* < 0.01), and the expression of Aggrecan and Sox9 genes distinctly increased at the concentration of 3.125 mg/mL and 6.25 mg/mL (*p* < 0.05). These results indicated that the L2C4S4 had conducive stimulatory effects on the chondrogenic differentiation of iPSCs.

### Low level of hypertrophy in the iPSC-chondrogenic spheres cultured in extracts of L2C4S4

**Fig. 6:**
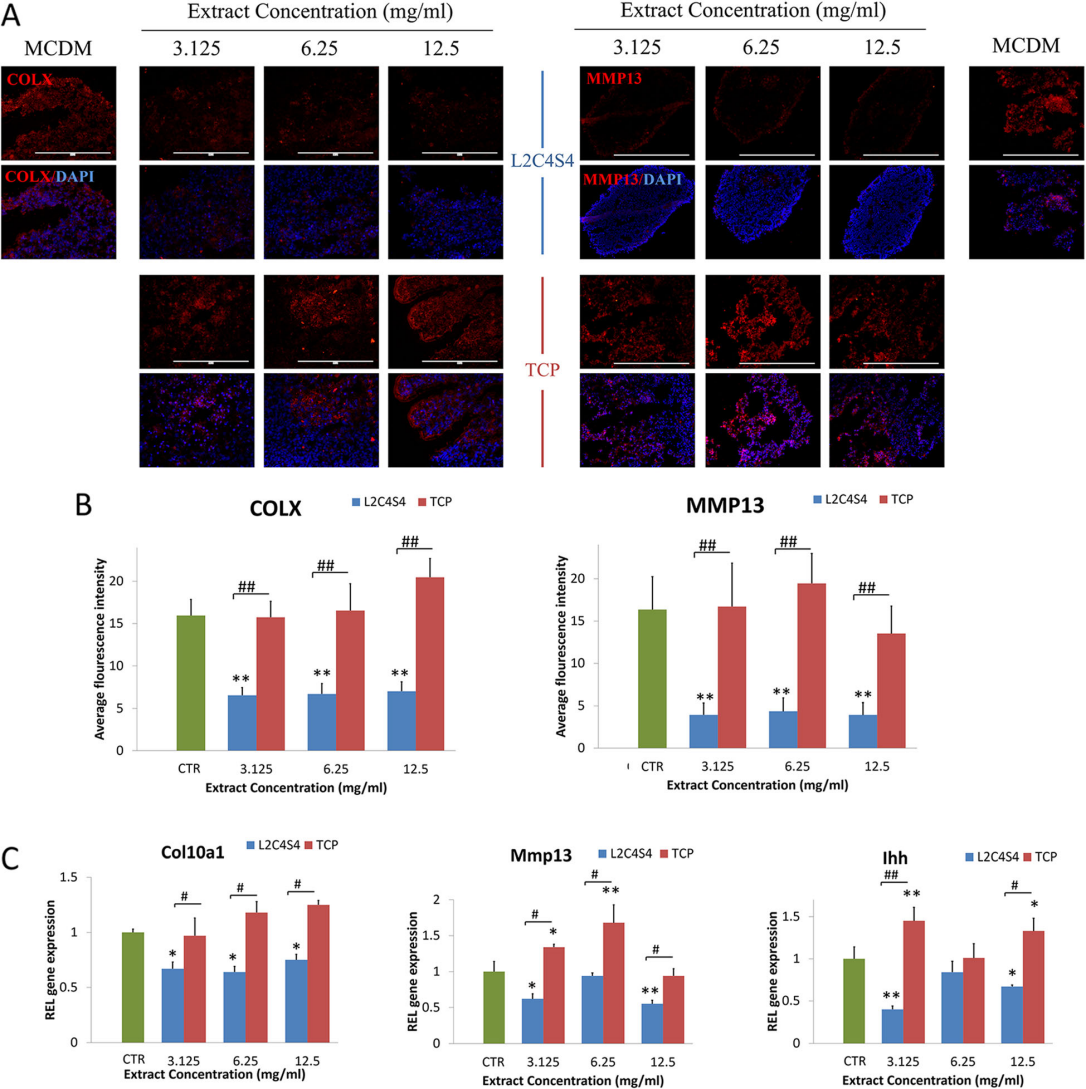
The hypertrophic differentiation of iPSC-derived chondrocytes with different concentrations of L2C4S4/ TCP extracts in 14 days. **a** Immunofluorescence of hypertrophic specified protein COLX and MMP13. **b** The average fluorescence intensity of COL X and MMP13 protein in each group, *n* = 5. **c** RT-PCR of hypertrophic genes Col10a1, Mmp13, and Ihh, *n* = 3. Data presented as mean ± SEM. Scale bar = 200 μm. CTR: MCDM without any extracts. ^#^*p*< 0.05; ^##^*p* < 0.01, *p < 0.05, **p < 0.01

In order to better determine the differentiation status of chondrocytes, chondrocyte hypertrophy markers COL X and MMP13 were analyzed after 14 days of iPSCs-chondrogenic differentiation. The results from immunofluorescence showed an obvious reduction of COL X and MMP13 in L2C4S4-treated cells than in TCP-treated cells and control group at different concentrations of extracts (3.125~12.5 mg/mL, *p* < 0.01, Fig. 6a). The average fluorescence intensity of COL X and MMP13 proteins in the control group and TCP-treated cells was more than twice as high as the L24S4-treated cells (Fig. 6b, *p* < 0.01). Hypertrophic-related genes COL X, Mmp13 and Indian hedgehog signaling molecule (Ihh) were also evaluated by RT-PCR in each group. TCP-treated cells and control group had greater expression of these three genes than L2C4S4-treated cells (*p* < 0.05, Fig. 6c). Thus, extracts of L2C4S4 could effectively reduce the level of hypertrophy in the iPSC-chondrogenic spheres.

### The accelerated effect of Li^+^ ions on the chondrogenic differentiation of iPSCs

**Fig. 7:**
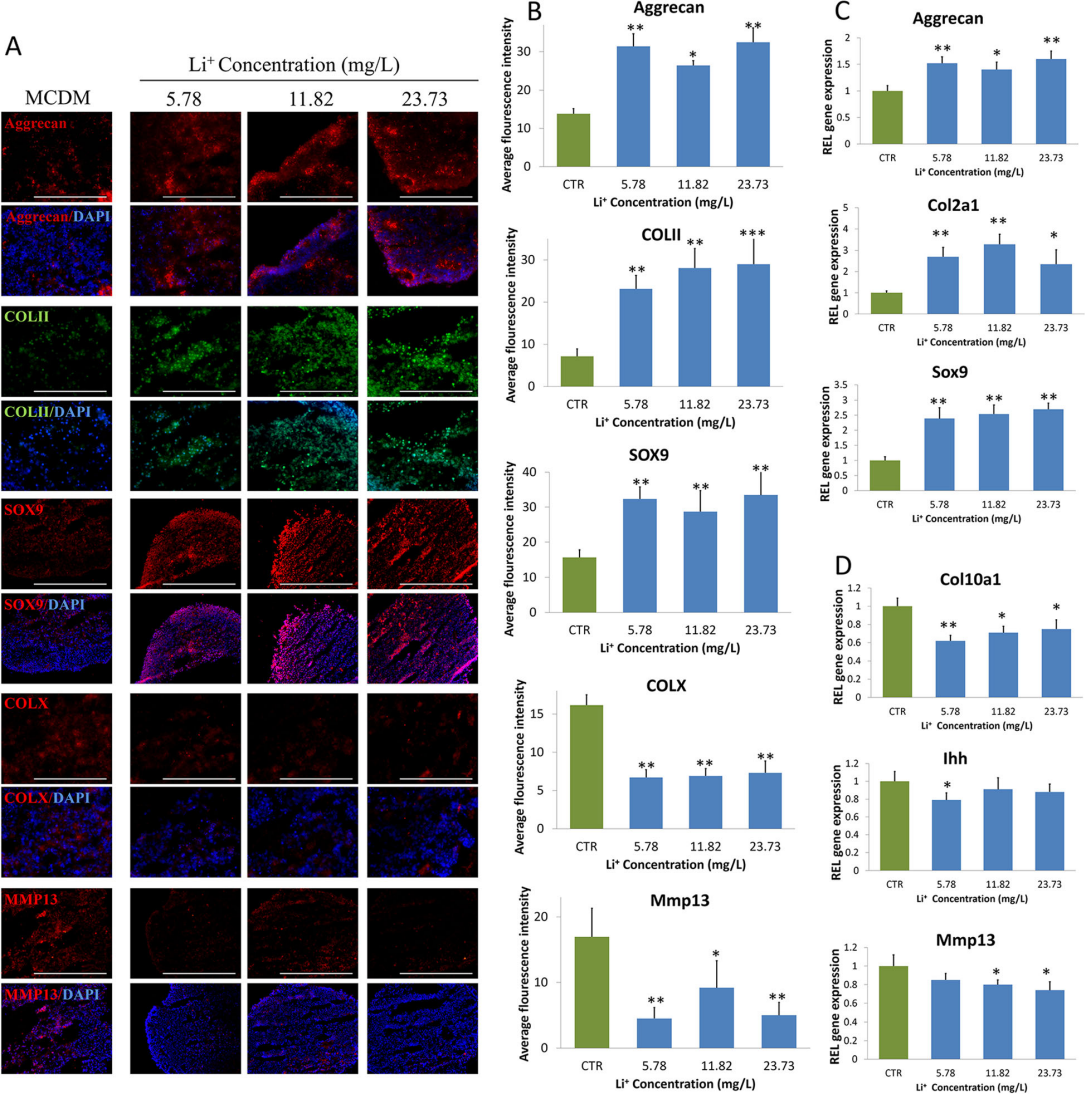
The chondrogenic differentiation of iPSCs with different Li^+^ ions concentration in 14 days. **a** Immunofluorescence of chondrocytes specified proteins (COL II, Aggrecan, and SOX9) and hypertrophic specified protein (COL X and MMP13). **b** The average fluorescence intensity of COL II, Aggrecan, SOX9, MMP13, and COL X proteins in each group, *n* = 5. **c** The relative genes expression of chondrogenic differentiation cultured with Li^+^ ions, *n* = 3. **d** The relative genes expression of hypertrophic differentiation cultured with Li^+^ ions, *n* = 3. Data presented as mean ± SEM. Scale bar = 200 μm. CTR: MCDM without any Li^+^ ions. *p < 0.05, **p < 0.01, ***p < 0.001

In order to seek out which ingredient of L2C4S4 that played a crucial part in chondrogenic differentiation, the ionic concentrations of calcium (Ca), lithium (Li), and phosphorus (P) ions in graded extracts of L2C4S4 and TCP were calculated by inductively coupled plasma atomic emission spectrometry. The values were given in Table 1. There were no significant differences between Ca and P ions in the extracts of L2C4S4 and TCP. In contrast, the Li^+^ ions concentration was positively correlated with the concentration of the L2C4S4 extracts. Corresponding to the concentration gradient of different L2C4S4 extracts (3.125~12.5 mg/mL), the concentration of Li^+^ ions varies within 5.78~23.73 mg/L. In addition, there were no Li^+^ ions existing in TCP extracts and MCDM. Based on these data, we decided to verify the effects of Li^+^ ions on chondrogenic differentiation of iPSCs. To mimic Li^+^ ions concentrations in L2C4S4 extracts, 5.78~23.73 mg/L Li^+^ ions were prepared of LiCl and applied in MCDM to culture iPSCs. MCDM without any extracts was served as a control. In immunofluorescence analysis, the fluorescence intensity of chondrocyte proteins COL II, Aggrecan, and SOX9 in Li^+^ ions-treated cells was all stronger than MCDM-treated cells apparently; meanwhile, the expression of hypertrophic marker COL X and MMP13 showed the opposite trend (Fig. 7a).

**Table 1:** The ionic concentrations of Ca, Li and P ions in graded extracts

Medium	Iron concentration		
	Ca (mg/L)	Li (mg/L)	P (mg/L)
MCDM	85.24	–	17.68
L2C4S4 + MCDM (3.125 mg/mL)	84.32	5.78	17.42
L2C4S4 + MCDM (6.26 mg/mL)	83.57	11.82	17.12
L2C4S4 + MCDM(12.5 mg/mL)	82.34	23.73	16.52
TCP + MCDM (3.125 mg/mL)	85.14	–	18.17
TCP + MCDM (6.26 mg/mL)	84.34	–	18.49
TCP + MCDM (12.5 mg/mL)	82.46	–	19.03

*MCDM* serum-free chondrogenic differentiation medium

The average fluorescence intensity of COL II, Aggrecan, and SOX9 proteins in Li^+^ions-treated cells was more than twice as high as the control group (Fig. 7b, *p* < 0.05). The expression of chondrogenic genes Col2a1, Aggrecan, and Sox9 all rose in different concentrations of Li^+^ ions-treated cells compared with the control group (*p* < 0.05, Fig. 7c). On the contrary, the expression of the Col10a1 gene in Li^+^ ions-treated cells at the concentration range of 5.78~23.73 mg/L was far less than the control group (*p* < 0.05, Fig. 7d). The Ihh gene was significantly reduced by Li^+^ ions at the concentration of 5.78 mg/L (*p* < 0.05) and the expression of the Mmp13 gene distinctly decreased at the concentration of 11.82 mg/L and 23.73 mg/L Li^+^ ions (*p* < 0.05, Fig. 7d). These data confirmed that individual Li^+^ ions at different concentrations (5.78~23.73 mg/L) also accelerated the chondrogenic differentiation of iPSCs and significantly prevented their hypertrophy.

## Discussion

In previous studies, a variety of biomaterials exhibited good effects on the maintenance of cartilage [11–15]. However, whether a biomaterial can observably promote chondrogenic differentiation of human iPSCs in vitro has not reached a firm conclusion. The toxic effect of biomaterials on iPSCs is our first consideration. Owing to the good affinity of the L2C4S4 for rabbit MSCs in our previous studies, we also tried to apply same dilutions of extracts (3.125~12.5 mg/mL) of L2C4S4 into iPSCs culture and observed vigorous growth cells with high pluripotency, which was of great significance for the subsequent differentiation process.

Well-formed chondrocyte spheres are of profound significance for cartilage defect repair [16]. Although the technology for applying biomaterials and iPSCs to cartilage tissue engineering was becoming increasingly sophisticated [17, 18], the shape and morphology of iPSCs-derived chondrocyte spheres were often overlooked. According to our results, it indicated that the use of L2C4S4 extracts can help maintain the good shape and suitable volume of iPSCs-derived chondrocyte spheres, which were roughly equivalent to spheres obtained by other induction methods [19, 20].

**Fig. 8:**
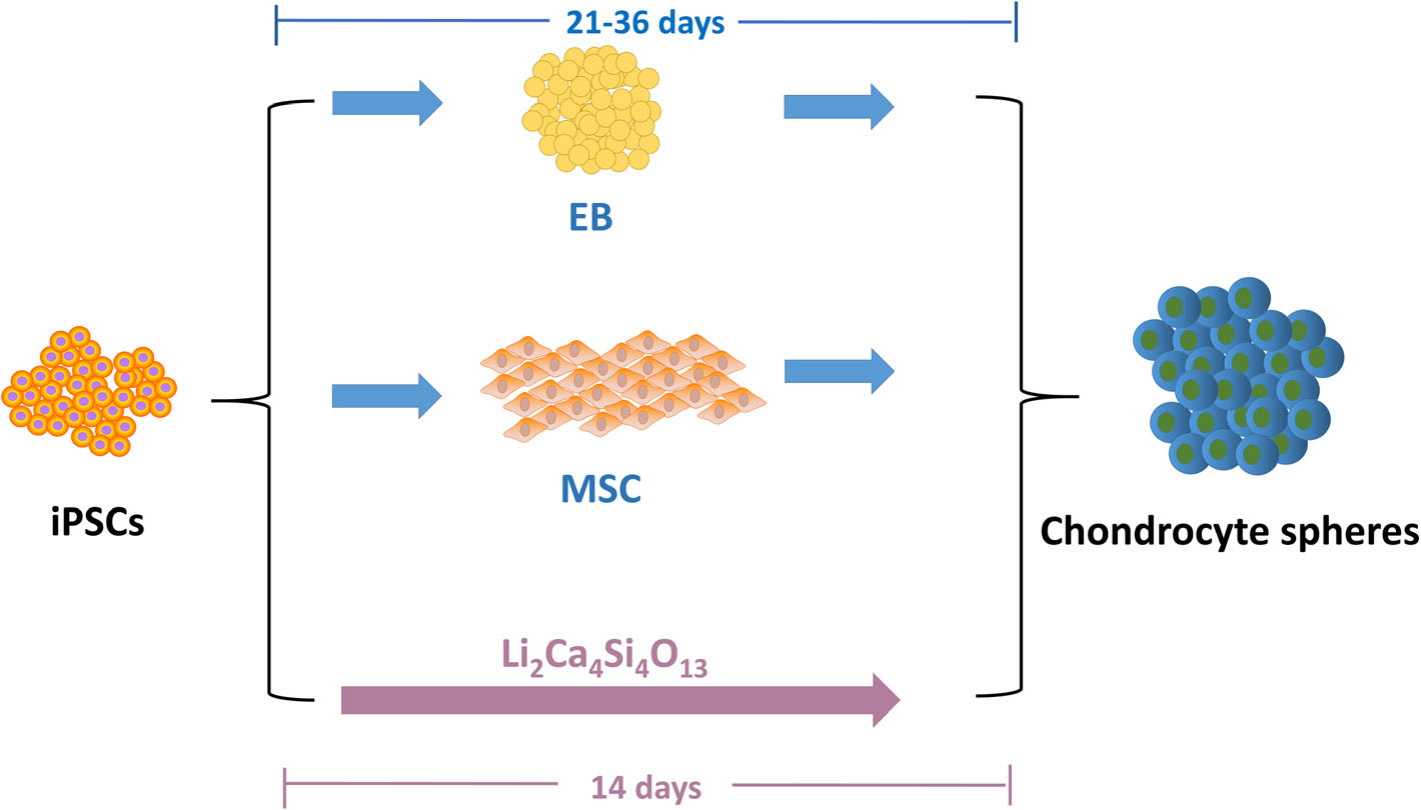
The chondrogenic differentiation strategies of iPSCs

COL II and Aggrecan are key chondrocyte proteins enriched in the cartilage matrix and withstand compression in cartilage. Sox9 is the master transcription factor of chondrogenesis and acts during chondrocyte differentiation. The substantial synthesis of these three markers meant that the iPSCs could be induced into mature chondrocytes with the help of L2C4S4 extracts. Although iPSCs could be induced into chondrocytes in various ways [6, 21, 22], it still existed a little regret of time-consuming in these methods. However, our results revealed a vital function of L2C4S4 in promoting the rapid conversion of iPSCs to mature chondrocytes within only 14 days. It was a one-step differentiation method and quicker than common approaches (Fig. 8). Regardless of the fact that the induction time was greatly shortened with the help of L2C4S4 extracts, the SOX9, and Aggrecan synthesized by the new-derived chondrocytes are comparable to those from other induction methods [23, 24].

Articular chondrocyte prehypertrophic differentiation is essential for osteoarthritis initiation and progression [25]. Hypertrophic chondrocytes may die or survive the cartilage-to-bone transition and become osteogenic cells in endochondral bones [26]. Avoiding cartilage hypertrophy is a key challenge when repairing articular cartilage. However, the evaluation of cartilage hypertrophy was often ignored in cartilage tissue engineering [27–29]. COL X is a short-chain collagen expressed by hypertrophic chondrocytes during endochondral ossification and often used as a classic marker of hypertrophic chondrocytes. Mmp13 is a member of matrix metalloproteinases family and involves in the breakdown of the extracellular matrix in cartilage. Our L2C4S4 biomaterial demonstrated superior hypertrophy inhibition in iPSCs-derived chondrocytes as to lower COL X and MMP13 expression. Ihh is a member of the hedgehog family and plays a role in bone growth and differentiation. This gene is also considered as important markers in hypertrophic chondrocytes. L2C4S4 extracts significantly reduced its expression in chondrocytes which indicated a lower level of hypertrophy in the iPSC-chondrogenic spheres. It revealed that these spheres were more suitable for repairing articular cartilage defects.

The most significant difference of ionic composition between L2C4S4 extracts and MCDM was Li^+^ ions concentration. Li^+^ ions were proved to have significantly admirable effects on cartilage [30], such as enhancing glycosaminoglycan-rich cartilage matrix production and protecting cartilage from degradation [31, 32]. However, the role of Li^+^ ions on iPSCs has been rarely discussed. Compared to these studies, our results further showed that Li^+^ ions could promote iPSCs to rapid chondrogenic differentiation and inhibit their hypertrophy. It was most likely that Li^+^ ions in L2C4S4 played the principal role. As previous studies have shown that Li-releasing biomaterials could induce chondrogenic differentiation, hyaline cartilaginous matrix formation [33] and enhance cartilage regeneration [34], our L2C4S4 bioceramics is also expected to be applicable to clinical cartilage repair due to the good release capacity of Li^+^ ions.

## Conclusions

It is the first attempt to apply L2C4S4 bioceramics into chondrogenic differentiation of human iPSCs with a distinct function. In our study, the introduction of L2C4S4 bioceramic not only promoted the expression of COL II, Aggrecan, and SOX9 but also reduced COL X/MMP13 production in iPSC-derived chondrocytes. The L2C4S4 extracts at the concentration range of 3.25~12.5 mg/mL distinctly stimulated the fast chondrocyte differentiation of iPSCs and inhibited their final hypertrophy. Furthermore, the stimulatory effects of Li^+^ ions in chondrogenic differentiation of iPSCs showed similar results, which proved that Li^+^ ions in L2C4S4 extracts may play a major role in the differentiation process. These results revealed that L2C4S4 bioceramic can biologically meet the demands of rapid chondrogenesis of human iPSCs and may serve as a useful platform for cartilage repair in the future. This study offered a viable new strategy for articular cartilage regeneration from an adequate source.

## Supplementary information

**Additional file 1:.** Table S1. Designations, sequences, and the sizes of RT- PCR amplicons.

## Data Availability

All supporting data are included in the article and its additional files.

## Abbreviations

iPSCs: Induced pluripotent stem cells
L2C4S4: Lithium-containing bioceramic
COL II: Type II collagen
COL X: Type X collagen
MSCs: Mesenchymal stem cells
EBs: embryoid bodies
Mmp13: Matrix metallopeptidase 13
SOX9: SRY-Box 9
Ihh: Indian hedgehog signaling molecule
Ca: Calcium
Li: Lithium
P: Phosphorus
RT-PCR: Real-time quantitative polymerase chain reaction
TCP: Tricalcium phosphate
